# Impact of a Student-Driven, Virtual Patient Application on Objective Structured Clinical Examination Performance: Observational Study

**DOI:** 10.2196/jmir.7548

**Published:** 2018-02-22

**Authors:** David Bergeron, Jean-Nicolas Champagne, Wen Qi, Maxime Dion, Julie Thériault, Jean-Sébastien Renaud

**Affiliations:** ^1^ Faculté de médecine Université Laval Québec, QC Canada; ^2^ Département de mathématiques et statistiques Université Laval Québec, QC Canada

**Keywords:** peer-assisted learning, virtual patient, medical education, undergraduate

## Abstract

**Background:**

Peer-assisted learning (PAL) refers to a learning activity whereby students of similar academic level teach and learn from one another. *Groupe de perfectionnement des habiletés cliniques* (Clinical Skills Improvement Group), a student organization at Université Laval, Canada, propelled PAL into the digital era by creating a collaborative virtual patient platform. Medical interviews can be completed in pairs (a student-patient and a student-doctor) through an interactive Web-based application, which generates a score (weighted for key questions) and automated feedback.

**Objectives:**

The aim of the study was to measure the pedagogical impact of the application on the score at medical interview stations at the summative preclerkship Objective Structured Clinical Examination (OSCE).

**Methods:**

We measured the use of the application (cases completed, mean score) in the 2 months preceding the OSCE. We also accessed the results of medical interview stations at the preclerkship summative OSCE. We analyzed whether using the application was associated with higher scores and/or better passing grades (≥60%) at the OSCE. Finally, we produced an online form where students could comment on their appreciation of the application.

**Results:**

Of the 206 students completing the preclerkship summative OSCE, 170 (82.5%) were registered users on the application, completing a total of 3133 cases (18 by active user in average, 7 minutes by case in average). The appreciation questionnaire was answered online by 45 students who mentioned appreciating the intuitive, easy-to-use, and interactive design, the diversity of cases, and the automated feedback. Using the application was associated with reduced reported stress, improved scores (*P*=.04), and improved passing rates (*P*=.11) at the preclerkship summative OSCE.

**Conclusions:**

This study suggests that PAL can go far beyond small-group teaching, showing students’ potential to create helpful pedagogical tools for their peers.

## Introduction

Peer-assisted learning (PAL), whereby students of similar academic level teach and learn from one another during a structured activity, is becoming increasingly popular in medical schools worldwide [1]. PAL benefits both the student-teachers, who develop communication skills and consolidate their knowledge, and student-learners, who benefit from a safe learning environment and cognitive congruence with teachers who better understand their learner’s perspective [2,3]. An area where PAL is particularly beneficial is the practice of medical interview and physical examination, for which autonomous study is insufficient and active learning with peers is essential [4,5]. One way by which students practice their interview and examination with peers is by presenting fictional cases to each other in the context of a mock Objective Structured Clinical Examination (OSCE) [6,7] or simply in a study group.

Thanks to advances in technology, the use of virtual patients (VPs) has a growing place in medical curricula worldwide [8,9]. VPs are a specific type of computer program that simulates real-life clinical scenarios for the purpose of medical education and assessment [8]. Although VP sharing initiatives like the Canadian Healthcare Education Commons– *Collaboration pour l’éducation en santé au Canada* Virtual Patient Working Group of the Association of Faculties of Medicine of Canada [10] exist, VPs are currently expensive to produce on a large scale (8). Medical students, born in the technological era, represent an undervalued resource to foster VP development through PAL.

As an attempt to bring PAL into the technological era, the *Groupe de perfectionnement des habiletés cliniques* (GPHC; translation: Clinical Skills Improvement Group) at Université Laval, Canada, created a collaborative VP platform containing over 220 peer-reviewed clinical cases. Cases were made available through an interactive Web-based application that allows students to practice their medical interview, receive automated feedback, and track their progression within their personalized profile. When launched in March 2016, the GPHC VP application was very well-received and used by most students for the OSCE study period. In this manuscript, we describe the development of the VP application and present a validation study on its impact on students’ performance at the final preclerkship OSCE. We hypothesized that using the application would increase the likelihood of having a passing score at the OSCE and that the extent to which it is used (number of cases) would correlate positively with OSCE final score.

## Methods

### Development of a Web-Based Virtual Patient Application

The GPHC is a PAL organization formed by preclerkship medical students at Université Laval. Founded in 2010, the GPHC helps peers improve their clinical reasoning and physical examination skills by organizing PAL workshops and mock OSCEs. In addition to workshops, the GPHC collaborates with faculty members to develop pedagogical material, including the *Petit Guide des Habiletés Cliniques* (translation: Pocket Guide to the Physical Examination) and the *Petit Guide de l’Entrevue Médicale* (translation: Pocket Guide to the Medical Interview), summarizing the key aspects of the medical interview and physical examination to help students preparing for their OSCE. To complement these books, the GPHC sought to create a digital platform that facilitates the practice of the medical interview.

With the financial support from Université Laval medical students’ investment funds, the GPHC hired 2 students in software engineering to develop a Web-based VP platform. In parallel, the GPHC launched a large-scale case writing campaign, through which over 100 medical students helped create 230 clinical scenarios. Students had to cite the references used to create their clinical scenario. Each case underwent a 2-level peer-review process—first from the student-author of the corresponding chapter of the pocket guide to the medical interview and then from a member of GPHC’s executive committee. We collaborated with software engineers to produce an interactive Web-based application interface that would adapt to every device (computers, phones, tablets) and provide a score, automated feedback, and graphs to measure progression. At present, the application is used at Université Laval, Université de Montréal, McGill University, and Ottawa University, with 1150 active users.

**Figure 1:**
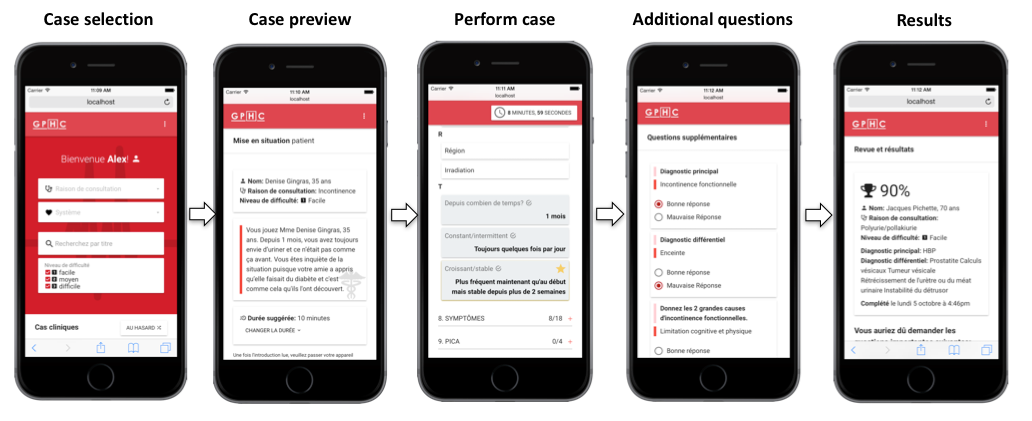
Interface of the Web-based application.

The application is designed to be used in pairs—by a student-patient and a student-doctor—in the following sequence (see Figure 1 for a summary): students first select 1 of the 230 cases based on a difficulty level (easy, moderate, hard), system (cardiology, pneumology, etc), or complaint (cough, nausea, fever, etc); for additional challenge, a random mode is available. Students can then set the time allocated to complete the case. Once a case is chosen, a case summary is presented to the student-patient, who can also preview the full questionnaire to analyze how the case is structured. In all clinical scenarios, questions are ordered in the prototypical, structured manner that is taught in classrooms. Then, a case statement is formulated to the student-doctor: “M. Gagnon, 56 years old, presents for diarrhea. Proceed to the questionnaire to determine the most likely cause of his symptoms.”

When the student-patient starts the simulation, the timer starts ticking and the student-doctor has a limited time to ask questions (can be set at 6, 8, 10, or 12 minutes). When questions are asked, the patient clicks on the question on the application and the answer appears. The application records each question asked. When time is over, the student-doctor is asked additional questions: “What is the most likely diagnosis?” “Can you name 3 probable alternate diagnoses?” and 2 additional questions (usually on management or physiopathology). The application then generates a score based on the questions asked, weighted for key questions.

In each scenario, 10 questions are rated as most important by the case-writer and reviewers, either because they give a good lead on the differential diagnosis or because they are essential to eliminate an imminent dangerous condition. Some aspects of communication, concerning, for instance, the patient’s agenda (concerns, ideas, and expectations), were included as key questions in some cases. The total score is based on the following: 50% for key questions (5% each), 15% for primary diagnosis, 15% if differential diagnosis was adequate, and 20% for other nonkey relevant questions (eg, allergies, habitus, review of systems), with a 5% bonus for 2 additional questions. This score calculation was chosen to reflect clinical reasoning over reciting a list of undifferentiated questions. The application also generates an automated feedback, summarizing which key questions were asked or forgotten, reinforcing the student’s ability to execute a medical interview.

After the case is finished, students are asked to rate their appreciation of the case and formulate comments (eg, flag errors, recommend other alternate diagnoses for the list) so that case quality is improved over time by revision from GPHC’s executive committee. Scores and feedback for every completed case are kept in a personalized profile where students can track their progression (score to each case, cumulative average), compare to other users (percentile for each case, each system, and overall), and consult previously completed cases. Finally, frequent users are offered the opportunity to give back to their peers by writing new cases, which undergo peer review before being added to the case list.

### Validation Study Design

Université Laval’s preclerkship medical program can be completed in 2, 2.5, or 3 years. At the end of the curriculum, students must pass a summative OSCE to continue toward their clerkships. On May 22, 2016, 206 students underwent their preclerkship summative OSCE. Of these students, 170 (82.5%) were registered users on GPHC’s application. We compiled every case (duration >3 minutes) performed by the students during the 2 months preceding the OSCE (3133 cases total; 18 per student on average). Students were informed that data collected in the application could be used for research purposes. We divided cases into 5 periods: period 1 (week preceding the OSCE, May 15 to May 21), period 2 (the preceding week, May 8 to May 14), period 3 (the preceding week, May 1 to May 7), period 4 (the preceding week, April 24 to April 30), and period 5 (the 3 preceding months, January 1 to April 23).

We calculated the number of cases of >3 minutes completed by each student in each period as well as their average score in each period. With the approval of the medical program and ethics review board, we accessed the results of the summative OSCE stations. We calculated a mean of the medical interview stations. Finally, we produced an online appreciation form asking students to rate statements on a 5-point Likert-type scale (from 1=strongly disagree to 5=strongly agree) and comment about what they like about the application. We analyzed answers to open questions using inductive thematic analysis [11]. Primary outcomes were student’s OSCE score and likelihood of passing preclerkship OSCE. Secondary outcomes were correlation between application use and OSCE score, increase in application scores over time, and thematic analysis of the online appreciation form. The research protocol was accepted by Université Laval ethics review board (*Comité d’éthique de la recherche avec des êtres humains de l’Université Laval*).

### Statistical Analyses

All statistical analyses were performed in SAS 9.3 (SAS Institute Inc). Significance level was set at *P*<.05. We dichotomized the score at final preclerkship OSCE between the student who passed the medical interview section of the OSCE (score ≥60%) and failed it (score <60%). We also dichotomized students based on their use of the application (≥10 versus <10 completed cases). The cutoff of 10 cases was selected after initial analysis of the data to allow for balanced group size. We used logistic regressions to measure whether completing ≥10 cases on the application increased a student’s likelihood of the passing preclerkship OSCE. We used a 1-sided Student *t* test to determine whether students who completed ≥10 cases had higher scores than those who completed <10 cases. We also used linear regressions to measure the association between use of the application (total number of cases completed) and the mean score on the application and the final preclerkship OSCE. We calculated a 95% confidence interval of *R*^2^ determination coefficient.

## Results

For the 206 students who underwent their preclerkship summative OSCE, 182 (88.3%) passed the medical interview section and 24 (11.7%) failed. During the study period, 3133 cases were completed by the 170 users (18.4 per student on average). Most cases (2224/3133, 70.99%) were performed in the 2 weeks preceding the summative OSCE (see Table 1). Average scores steadily improved from 69.8% in period 4 (April 24 to April 30) to 80.5% in period 1 (May 15 to May 21, summative OSCE being on May 22). A slight decrease was observed between period 5 (74.1%) and 4 (69.8%).

**Figure 2:**
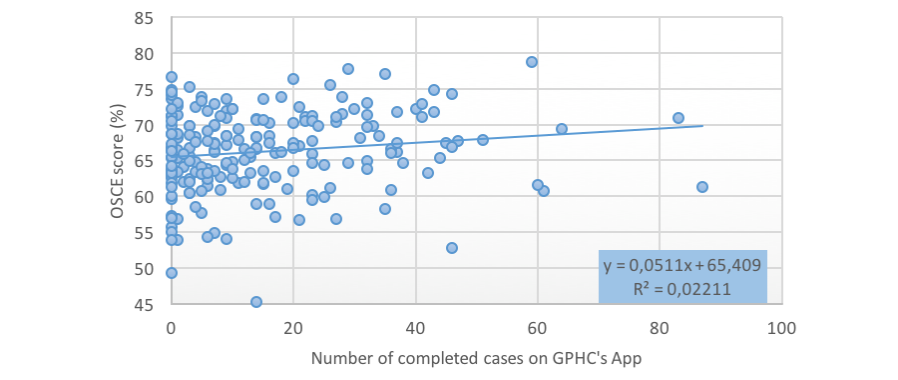
Relationship between number of completed cases on the application and the Objective Structured Clinical Examination score (OSCE).

Frequent users of the application (≥10 cases completed) had significantly higher scores (66.9% SD 5.5) on the OSCE medical interview sections than those who completed <10 cases (65.5% SD 5.6, *P*=.04). They also trended toward a higher likelihood of having a passing grade in the medical interview sections (90.3%) than those who completed <10 cases (86.3%, *P*=.11, not significant). Total number of cases completed had a low correlation with the OSCE medical interview score (*R*^2^=.02, 95% CI –0.01 to 0.284; see Figure 2).

The appreciation questionnaire was answered online by 45 students (see Table 2). Students who answered the questionnaire were for the most part frequent users of the application (91% with >10 completed cases, 62% with >20 cases). The questions reaching the greatest consensus were “I prefer to use only the pedagogical material provided by professors because I do not trust the quality of material produced by peers” (median 2) and “I feel that practicing with GPHC’s application has helped me to better structure my anamnesis” (median 5). In their comments, convergent themes revealed by inductive thematic analysis were the intuitive, easy-to-use, and interactive design, the diversity of cases, and the automated feedback (see Textbox 1).

**Table 1:** Use of the application throughout the study period.

Period	Dates	Cases completed, n	Active users, n	Cases by active users, mean (SD)	Average score, mean
5	January 1 to April 23	79	17	4.9 (7.3)	74.1
4	April 24 to April 30	109	26	4.4 (3.2)	69.8
3	May 1 to May 7	729	91	8.1 (6.4)	75.2
2	May 8 to May 14	962	125	7.8 (6.1)	76.0
1	May 15 to May 21	1254	130	9.7 (7.6)	80.5
Total	January 1st to May 21	3133	170	18.5 (17.2)	79.7

**Table 2:** Results of the appreciation questionnaire.

Statement	Median^a^
I prefer to use only the pedagogical material provided by professors because I do not trust the quality of material produced by peers.	2
I prefer practicing the clinical examination by actively performing cases in the application to autonomous study in books.	5
I feel that practicing with the GPHC^b^application has helped me to better structure my anamnesis.	5
I feel that practicing with GPHC’s application has helped me to improve my clinical reasoning.	5
Practicing with GPHC’s application has improved my confidence regarding the summative OSCE^c^.	5
Practicing with GPHC’s application has reduced my stress level related to the summative OSCE.	4

^a^Score represent a 5-point Likert-type scale, from 1=strongly disagree to 5=strongly agree.

^a^GPHC: *Groupe de perfectionnement des habiletés cliniques.*

^a^OSCE: Objective Structured Clinical Examination.

**Comment sample from the appreciation questionnaire.**

Increase efficiency

- “The large bank of peer-reviewed clinical cases saves us the time we would take to write cases, that we can use more efficiently by doing more cases on the app!”
- “The app allows us to do more cases without having to write them ourselves, which increases the efficiency of our study.”
- “Interactive, lots of cases easily available, simple to use, I love it!”

Easy to use

- “The app allowed me to practice my OSCE with relatives who do not have a medical background.”
- “It is interactive, and we can even practice with people who do not study medicine!”

Get out of our comfort zone

- “The diversity of the cases helps us identify our weaknesses and exposes us to cases we would not write inside our study group.”
- “The random mode allows us to get out of our comfort zone and be prepared for a wide variety of cases.”

Weaknesses

- “...however, some cases still contain errors. Hopefully the feedback function will allow you to improve them over time.”

## Discussion

### Principal Findings

As a student-led PAL organization, we developed a collaborative VP platform to improve medical interview skills among preclerkship medical students. The Web-based interactive application was used by 82.5% (170/206) of students in their preparation for the summative preclerkship OSCE, who appreciated the intuitive, easy-to-use, and interactive design, the diversity of cases, and the automated feedback. Students who used the application reported reduced stress levels related to the preclerkship summative OSCE. We conclude that students can successfully create learning tools that potentially improve their peers’ performance at summative evaluations.

This initiative represents a unique contribution to the eLearning field. We believe that most current VP platforms are ill-suited to many aspects of the development of history-taking, examination, communication, and procedural skills of novice learners [12]. They usually consist of single-user clinical scenarios where the student has to interrogate the patient (computer) by selecting questions, physical examination maneuvers, and laboratory tests to perform and ultimately commit to a diagnosis and/or management plan. By displaying a list of questions and maneuvers, they prevent students from thinking of questions themselves, artificially relieving them from practicing an important part of the clinical examination. Indeed, one of the main difficulties novices face during clinical examination is cognitive overload, whereby they devote so much cognitive capacity to determining which questions to ask (or maneuvers to execute) that they have insufficient cognitive resources to simultaneously interpret findings in light of their differential diagnosis [13].

Our VP application, designed to be used in groups of 2 students (a student-patient and a student-doctor), places users in a much more realistic clinical environment, where they have to simultaneously determine which questions to ask and interpret answers. Another benefit of our student-run VP platform is its low cost. From a medical program perspective, developing a VP is currently costly, as 85% cost more than $10,000 per case and 37% cost more than $50,000 per case [14]. For a total cost of Can $23,000 (US $18,700) (plus $3,000 per year for system maintenance), the GPHC application is an autonomous collaborative platform containing over 220 VP scenarios.

Our VP application also represents a novel and important advance in the field of PAL. To our knowledge, this is the first peer-led VP platform in which students can create, revise, and use clinical scenarios. Most PAL initiatives reported to this date consist of small-group workshops and/or mock OSCE exams [1,3,6,7]. Using technology to foster collaboration, our application allows clinical cases created in a given study group to be used by all their colleagues and even future generations of students. The pedagogical material is self-improving through the contribution of users, who are asked to comment on existing cases (flagging errors, rating case quality) and submit new cases. Peer review of cases is performed by committee members of the PAL organization (GPHC). The result is a high-quality, self-improving VP platform helping students actively practice their medical interview and clinical reasoning.

### Limitations

Our study has limitations. First, motivation level represents a potential bias, as it influences both the use of the application and the use of traditional pedagogical tools (hence the OSCE score). Since we could not control for motivation level, we cannot exclude that the use of the application represents a sign of motivation and good studentship rather than a cause of good test results. Second, the effect of application use on OSCE scores, although significant at some statistical tests, remains modest. It was predicted from the start that application use would only marginally explain OSCE scores, as a variety of other factors are involved in test scores. Third, the fact that most students who answered the appreciation questionnaire were frequent users of the application may have led to an overestimation of positive answers. Likewise, reasons why nonusers have favored other study tools could not be evaluated. Nevertheless, the fact that the majority of students (83%) adopted the application as a significant study tool (18 cases per user on average) despite it being totally optional remains in our opinion the study’s most compelling result. Since they typically have a busy schedule and a wide variety of study tools at their disposition, medical students are very critical of the tools to which they will devote study time; their confidence in our student-led VP platform hence reflects its helpful complementarity with faculty-provided material.

### Conclusion

GPHC’s Web-based application is a student-run, collaborative VP platform. To our knowledge, it is the first digital PAL initiative of its kind. The application is highly appreciated among students, and its use was associated with increased scores at the summative preclerkship OSCE.

## Abbreviations

GPHC: Groupe de perfectionnement des habiletés cliniques
OSCE: Objective Structured Clinical Examination
PAL: peer-assisted learning
VP: virtual patient
